# Workgroup Report: Workshop on Source Apportionment of Particulate Matter Health Effects—Intercomparison of Results and Implications

**DOI:** 10.1289/ehp.7989

**Published:** 2005-09-01

**Authors:** George D. Thurston, Kazuhiko Ito, Therese Mar, William F. Christensen, Delbert J. Eatough, Ronald C. Henry, Eugene Kim, Francine Laden, Ramona Lall, Timothy V. Larson, Hao Liu, Lucas Neas, Joseph Pinto, Matthias Stölzel, Helen Suh, Philip K. Hopke

**Affiliations:** 1 Nelson Institute of Environmental Medicine, New York University School of Medicine, Tuxedo Park, New York, USA; 2 Department of Environmental Health, University of Washington, Seattle, Washington, USA; 3 Department of Statistics, and; 4 Department of Chemistry and Biochemistry, Brigham Young University, Provo, Utah, USA; 5 Department of Civil and Environmental Engineering, Southern California University, Los Angeles, California, USA; 6 Center for Air Resources Engineering and Science, Clarkson University, Potsdam, New York, USA; 7 Department of Environmental Health, Harvard School of Public Health, Boston, Massachusetts USA; 8 Department of Civil and Environmental Engineering, and; 9 Department of Biostatistics, University of Washington, Seattle, Washington, USA; 10 National Health and Environmental Effects Research Laboratory, U.S. Environmental Protection Agency, Chapel Hill, North Carolina, USA; 11 National Center for Environmental Assessment, U.S. Environmental Protection Agency, Research Triangle Park, North Carolina, USA; 12 Institute of Epidemiology, Focus Network Aerosols and Health, National Research Center for Environment and Health (GSF), Neuherberg, Germany

**Keywords:** fine particles, health effects, mortality, particulate matter, source apportionment, sulfate, time-series, uncertainty

## Abstract

Although the association between exposure to ambient fine particulate matter with aerodynamic diameter < 2.5 μm (PM_2.5_) and human mortality is well established, the most responsible particle types/sources are not yet certain. In May 2003, the U.S. Environmental Protection Agency’s Particulate Matter Centers Program sponsored the Workshop on the Source Apportionment of PM Health Effects. The goal was to evaluate the consistency of the various source apportionment methods in assessing source contributions to daily PM_2.5_ mass–mortality associations. Seven research institutions, using varying methods, participated in the estimation of source apportionments of PM_2.5_ mass samples collected in Washington, DC, and Phoenix, Arizona, USA. Apportionments were evaluated for their respective associations with mortality using Poisson regressions, allowing a comparative assessment of the extent to which variations in the apportionments contributed to variability in the source-specific mortality results. The various research groups generally identified the same major source types, each with similar elemental makeups. Intergroup correlation analyses indicated that soil-, sulfate-, residual oil-, and salt-associated mass were most unambiguously identified by various methods, whereas vegetative burning and traffic were less consistent. Aggregate source-specific mortality relative risk (RR) estimate confidence intervals overlapped each other, but the sulfate-related PM_2.5_ component was most consistently significant across analyses in these cities. Analyses indicated that source types were a significant predictor of RR, whereas apportionment group differences were not. Variations in the source apportionments added only some 15% to the mortality regression uncertainties. These results provide supportive evidence that existing PM_2.5_ source apportionment methods can be used to derive reliable insights into the source components that contribute to PM_2.5_ health effects.

Airborne particulate matter (PM) air pollution is presently regulated by the National Ambient Air Quality Standards (NAAQS) using gravimetric mass as the particle metric to assess air quality. However, an enormous number of different chemical species are associated with the various types of ambient particles, depending upon their source origins (e.g., Cooper and Watson 1980). For example, primary particles emitted from coal combustion are characteristically highly enriched with arsenic and selenium, whereas residual oil combustion particles are more enriched in nickel and vanadium, and soil particles are especially enriched in the crustal elements (e.g., silicon, aluminum). In addition, secondary components of particles (e.g., sulfates, nitrates, and organic compounds) are formed in the atmosphere from gaseous pollutant emissions. These secondary components can either condense on primary particles or form secondary particles that can then collide and coagulate with primary particles. Individual particles in an urban airshed can contain both primary and secondary components, and the composition of ambient aerosols have been found to reflect source PM emission characteristics differences over space (e.g., between cities) and time (e.g., across seasons) (e.g., Spengler and Thurston 1983). Because the composition of particle types varies greatly, it is probable that some types of particles are more toxic than others. Thus, treating all particles that contribute to the mass concentration equally in the regulatory process may lead to inefficient protection of public health. A potentially more effective regulatory approach would be to address the individual types of particles independently, focusing control efforts on the most toxic categories. However, because toxicities of individual source components are not yet certain, and because virtually all published PM health effects studies to date have used PM mass (in various size categories) as the particle pollution index, the current NAAQS for airborne PM use airborne particle mass as the indicator for making air quality compliance determinations. Equal treatment of all particles that contribute to mass, irrespective of composition, may be leading to less-optimal control strategies to avoid the adverse human health effects of PM, potentially causing the present PM ambient standard to be less protective of health in some areas of the nation than in others. There is a need for epidemiologic and toxicologic evaluation of the extent to which the toxicity of ambient PM mass varies by particle type and source.

Because source composition and/or physical properties of particles vary between different source categories, the mass can be statistically apportioned into contributions from various source categories, opening the possibility of evaluating PM component effects using epidemiologic methods presently used on the PM mass. As discussed by Hopke et al. (in press), this area of research, called receptor modeling, has been active for over 3 decades. A number of accepted methods are being used to apportion the total mass into source categories, and these source apportionment methods can now be used as inputs to epidemiologic models of the human health effects of air pollution. However, to date only a small number of published efforts have related source-apportioned PM impacts to human health effects (e.g., Laden et al. 2000; Mar et al. 2000; Ozkaynak and Thurston 1987). The effect of the imputation of these apportionments on the ability of epidemiologic methods to evaluate the health effects associated with various PM components is uncertain. Because a number of methods are used to determine source contributions to PM mass impacts, and application of these methods varies among researchers, their application, although providing new insights, can also be expected to introduce added uncertainty into the derivation of estimates of PM toxicity [e.g., to the estimation of mortality relative risks (RRs) per amount of mass of fine particulate matter < 2.5 μm in aerodynamic diameter (PM_2.5_)]. The scientific and regulatory community is uncertain whether meaningful and reliable source apportionments of PM_2.5_ health effects are possible with today’s data and methods. A workshop was therefore organized by a consortium of U.S. Environmental Protection Agency (EPA) PM centers to assess the extent to which variations in current source apportionment methods and their application may affect the ability of epidemiologic studies to discern PM health effects on a source-specific basis.

On 29–30 May 2003, the U.S. EPA PM centers sponsored the Workshop on the Source Apportionment of PM Health Effects, hosted by the New York University (NYU) PM Research Center. The specific goal of this workshop was to evaluate the variability of the various PM source apportionment approaches in assessing PM source contributions to ambient PM_2.5_ concentrations in real-world data sets and to then assess the influence of this variability on the ability of statistical time-series analyses to discern which source categories contribute significantly to daily PM_2.5_ mass–mortality associations. No new health or environmental data were generated by participants during this effort. Instead, the same pre-existing reference PM mass and constituent data sets from two cities (Washington, DC, and Phoenix, AZ) were sent to various leading source apportionment research groups in advance of the workshop (in December 2002), and each group individually analyzed the same data sets for daily source PM_2.5_ contributions. These various daily PM_2.5_ mass source apportionments were then independently submitted before the workshop (in April 2003), and each was individually evaluated for their respective associations with daily mortality in each city in a consistent manner across the various apportionment research groups/methods. The PM–mortality health effects time-series modeling evaluations were conducted for the Washington and Phoenix data sets by researchers at the NYU and University of Washington U.S. EPA PM Research Centers, respectively. Washington and Phoenix were selected for this workshop analysis because the PM data available from these cities in past years were collected and analyzed for trace constituents in a manner similar to that used by the U.S. EPA in the nationwide Speciation Trend Network (STN), so as to come to workshop conclusions relevant to that developing STN data set. In addition, the consideration of these two very different cities with differing sources and weather provided a broader test of the consistency of these methods than would a single city or two cities from the same region of the country. Keeping the health effects model consistent across the various source apportionment researchers and methods allowed a separate discernment of the extent to which variability in the source apportionment step contributed to variability in the ultimate health effects analyses results.

The goals of the workshop were to bring together key researchers to assess the reliability of source apportionment–health effects methods by analyzing daily mortality with existing PM_2.5_ data sets similar to those now being collected by the U.S. EPA Speciation Network and to identify key future research needs for source apportionment health effects evaluation. As noted in Table 1, research groups from seven institutions, using various source apportionment approaches, participated in this workshop. Most of the groups were affiliated with one of the five U.S. EPA PM centers.

## Materials and Methods

### Particulate matter data sets.

The two PM_2.5_ mass and composition data sets employed in the source apportionments were selected based on their ready availability for analysis, the similar availability of a compatible daily mortality record for health effects analysis, and the fact that their PM_2.5_ composition analyses were similar in many ways to those characteristics available to researchers from the new U.S. EPA PM_2.5_ STN. In this way, analyses of existing data sets could be accomplished quickly and would provide information relevant to future analyses that might be conducted with the rapidly expanding U.S. EPA STN database. Brief descriptions of these databases are provided below, and more detailed descriptions are provided in the companion workshop papers (Hopke et al., in press; Ito et al., in press; Mar et al., in press).

In Phoenix, daily integrated 24-hr samples were collected with a dual fine-particle sequential sampler (URG Corp., Chapel Hill, NC, USA) on 37-mm diameter Teflon and quartz filter media for fine particle mass and species measurements. A total of 981 samples were collected from March 1995 through June 1998. Each sample was characterized by the measured concentrations of the following 46 chemical elements: sodium, magnesium, aluminum, silicon, phosphorus, sulfur, chlorine potassium, calcium, scandium, titanium, vanadium, chromium, manganese, iron, cobalt, nickel copper, zinc, gallium, germanium, arsenic, selenium, bromine, rubidium, strontium, yttrium, zirconium, molybdenum, rhodium, palladium, silver, cadmium, tin, antimony, tellurium, iodine, cesium, barium, lanthanum, tungsten, gold, mercury, lead, organic carbon, and elemental carbon (EC). The analytical uncertainty estimates associated with each measured concentration and the detection limits for both instruments were also included.

In Washington the PM_2.5_ samples were collected on Wednesdays and Saturdays at the Interagency Monitoring of Protected Visual Environments (IMPROVE) monitoring site located in downtown Washington. A total of 718 samples were collected between 31 August 1988 and 31 December 1997. Integrated 24-hr PM_2.5_ samples were collected on Teflon, nylon, and quartz filters. The Teflon filters were used for mass concentrations and analyzed via particle-induced X-ray emission for the elements Na, Mg, Al, Si, P, S, Cl, K, Ca, Sc, Ti, V, Cr, Mn; via X-ray fluorescence for elements Fe, Co, Ni, Cu, Zn, Ga, Ge, As, Se, Br, Rb, Sr, Y, Zr, Mo, Rh, Pd, Ag, Cd, Sn, Sb, Te, I, Cs, Ba, La, W, Au, Hg, and Pb; and via proton elastic scattering analysis for elemental hydrogen concentration. The nylon filter was analyzed by ion chromatography for sulfate, nitrate, and chloride. The quartz filters were analyzed by the IMPROVE method for temperature-resolved organic and EC fractions (IMPROVE/TOR).

### Daily mortality data sets.

Washington death records were extracted from the National Center for Health Statistics database for the period from 31 August 1988 to 31 December 1997, and daily counts were aggregated for the District of Columbia and surrounding six areas: Montgomery County, Maryland; Prince George’s County, Maryland; Fairfax County, Virginia; and Alexandria, Fairfax, and Falls Church, Virginia. Three categories of deaths were analyzed: total nonaccidental; cardiovascular; and cardiovascular plus respiratory.

Phoenix mortality data from 1995 to 1997 were obtained from the Arizona Center for Health Statistics. In this analysis, we included only mortality counts for residents ≥65 years of age from ZIP code regions thought to be most represented by the U.S. EPA monitoring platform (Mar et al. 2000). We evaluated total nonaccidental mortality [*International Classification of Diseases, 9th Revision* (ICD-9) codes < 800.00; World Health Organization 1978] and cardiovascular mortality (ICD-9 codes 390.00–448.99) from 9 February 1995 to 31 December 1997. From 1995 to 1997 there were a total of 9,081 nonaccidental deaths and 4,109 cardiovascular deaths.

### Source apportionment modeling.

The above-described PM_2.5_ mass and composition data sets were provided to each participating research group in December 2002 for independent analysis, using each group’s preferred source apportionment technique(s). To allow a consistent intercomparison of results across research groups, participants were requested to submit results in a standardized format and with a list of items describing the details of source apportionment analysis (e.g., type and extent of rotation, treatment of outliers, criteria used to include species in the analysis). Of the 11 potential participants to whom the data were sent, eight participant/teams from seven institutions submitted source apportionment results by the required deadline (April, 2003).

As described in more detail in the companion paper by Hopke and collaborators (in press), the fundamental principle of source apportionment (receptor) modeling is that mass conservation can be assumed, and a mass balance analysis can be used to identify and apportion sources of airborne PM in the atmosphere. If the number and nature of the sources affecting the air-monitoring station are known, then the only unknown is the mass contribution of each source to each sample, *s_jk_*. These values can be estimated using regression. This approach was first independently suggested by Winchester and Nifong (1971) and Miller et al. (1972) and is now called the chemical mass balance (CMB) model (Chow and Watson 2002, Cooper and Watson 1980; Cooper et al. 1984). In general, CMB models assume that the recorded aerosol mass (*M_k_* ) in micrograms per cubic meter is due to the sum of impacts by individual sources (*S_jk_* ):


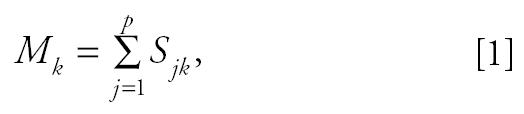


where *k* = 1,2, ....*m* days; *j* = 1,2, ....*p* sources, and the total concentration of aerosol property *C_ik_* (i.e., element *i* ’s ambient concentration on day *k* at a site) is


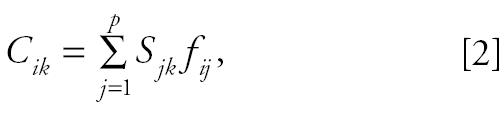


where *f_ij_* = the mass fraction of property *i* in emissions from source *j*.

Thus, if the source profiles (*f_ij_*) are known, the source contributions (*S_jk_*) can be determined from the linear regression of the *C_ik_* on the *f_ij_*.

However, if (as is more usually the case), the source emission “signatures” are not known exactly, but only qualitatively (e.g., that vanadium is enriched in residual oil combustion particles, but the exact percentage is not known), then factor analysis (FA) methods are applied to identify and quantify the sources and their impacts. The FA approach to source apportionment assumes that the total concentration of each observable (element) is made up of the sum of contributions from each of *p* pollution source components:


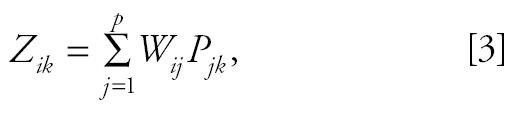


where


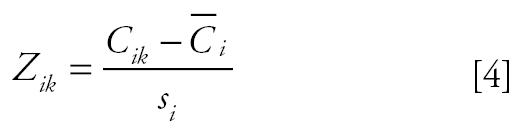


(the standardized *z*-score of element *i* ’s *k*th observation), and *P_jk_* = the *j*th factor component’s value on the *k*th day; *W_ik_* = the scoring coefficient matrix of the components; and *s_i_* = the standard deviation of element *i*.

With respect to CMB models, the *P_jk_* are equivalent to the *S_jk_* source impacts, and the *W_ij_* are equivalent to the *F_ij_* source profiles. However, the *P_jk_* and *W_ij_* are derived by the FA from the correlation matrix and are outputs of the FA (instead of inputs, as is the case for CMB). Such FA approaches generally have a major advantage, in that they can identify and quantify nontraditional aerosols such as secondary aerosols (formed in the atmosphere) and can incorporate non-PM tracers such as the gaseous pollutants. Such FA and principal components analysis (PCA) models attempt to simplify the description of a system by determining a minimum set of basis vectors that span the data space to be interpreted. In other words, a new set of variables is found as linear combinations of the measured variables so that the observed variations in the system can be reproduced by a smaller number of these causal factors. This approach has been widely used in studies of airborne PM composition data (Gao et al. 1994; Hopke et al. 1976; Roscoe et al. 1982).

Traditional FA and PCA are useful for identifying source components contributing to the PM mass but do not directly provide an apportionment in the form presented above. However, the solutions can be manipulated to provide such a quantitative solution. One approach is specific rotation FA (Koutrakis and Spengler 1987), which uses a targeted Procrustes factor rotation. An alternative approach, absolute principal-component analysis (APCA) (Thurston and Spengler 1985), has also been used to produce quantitative apportionments. Two more-recent approaches are Unmix (Henry and Kim 1999; Kim and Henry 1999, 2000a, 2000b) and positive matrix factorization (PMF) (Paatero 1997; 1999; Paatero et al. 2002). These and similar multivariate techniques, described and documented in more detail in Hopke et al. (in press), have been applied by the different research groups to achieve source apportionments of the Washington and Phoenix PM_2.5_ data sets (Table 2).

After all the estimated source-specific impact assessments were submitted by workshop participants, the agreement across source apportionment analyses was evaluated. This was first evaluated by an intercomparison of the various analyses’ respective mean estimates of source-specific mass impacts in each city. In addition, as the various source apportionment results were to be employed as inputs into a daily time-series mortality analyses, the time-series intercorrelations of their respective daily estimates of source impacts were also evaluated and intercompared across source categories in each city.

### Health effects modeling.

After the source apportionments were submitted, all Washington and Phoenix daily source apportionments were provided to K. Ito of the NYU PM Center and T. Mar of the University of Washington PM Center, respectively, for inclusion in time-series mortality models to assess the resulting variations in their source-specific health effects estimates (RRs). The city-specific mortality models employed are described below.

The model-building steps of the Washington time-series mortality model development used in these analyses (Ito et al., unpublished data) were designed to be similar to those used in past studies of PM_2.5_ mass, as follows:

- We first developed the base mortality model as a function of season and other temporal trends in Poisson generalized linear models (GLMs) (McCullagh and Nelder 1989). Using natural splines, we fit a smooth function of time to mortality to adjust the model for seasonal trends and unmeasured seasonal confounders, such as influenza epidemics. The inclusion of this term also reduces undesirable residual autocorrelation and overdispersion in the mortality regression, so the choice of the spline degrees of freedom (df) for smoothing of time (df = 38, or 4 per year) was based both on the fit to the mortality series and minimization of autocorrelation of the model residuals.
- Weather variables and a day-of-week variable were then also incorporated into the base model, consistent with past general practice in PM_2.5_ modeling, including *a*) natural splines of the same-day temperature with 4 df to fit “hot” temperature effects; *b*) natural splines of the average of lags 1 through 3 of daily temperature (i.e., up to 3 days before the date of death) to fit “cold” temperature effects; and *c*) an indicator for “hot” (daily mean temperature > 80°F) and “humid” (daily relative humidity > 70%) days to fit the interaction. The end result of this step was a base model to which air pollutant variables could be added and evaluated.
- To the base model, each of the alternative source components was individually added (for each research group/method) to separately test the individual associations of each source category with mortality, after controlling for the variables considered in the base model. The RR associated with both an interquartile (25th to 75th percentile) and a 5th- to 95th-percentile increase in the source estimate was computed for lag days 0 to 5 for each of the source apportionment analyses. This approach provided directly comparable mortality effect estimates for each source category and for apportionment modeling results of participating groups.

The basic steps of time-series model development used in the Phoenix analyses (Mar et al., in press) were the same as for Washington. Similarly, associations between source contributions and cardiovascular and total nonaccidental mortality were analyzed using Poisson GLMs in S-PLUS 2000 (Insightful Inc., Seattle, WA, USA). The same Phoenix base mortality model was applied to source apportionment analyses of all groups to provide a consistent basis for comparison across source components and groups (i.e., to eliminate model specification variability from the analysis). The base model controlled for extreme temperatures using an indicator variable, mean temperature, relative humidity, day of week, and time trends. Natural spline smoothers were used for time trends, temperature, and relative humidity. We applied 12 df for the smoothing of time trend (i.e., 4 df per year). The degrees of freedom for the natural splines for time trends were selected to minimize autocorrelation in the residuals and the Akaike Information Criterion (AIC) (Akaike 1974). For the analysis of cardiovascular mortality, 5 spline df and 2 days lag for temperature were incorporated, based on past experience with models of PM_2.5_ and mortality in this city. For the total mortality analysis, 5 spline df and 1 day lag for temperature were employed, and 2 df for the smoothing of relative humidity with 0 days lag for both the cardiovascular and total mortality analyses. The degrees of freedom and the lags were chosen to minimize the AIC. As in the case for the Washington analyses, the respective estimated source contributions of the various research groups were added to this base model, in turn, as the particle pollution variable. The RR associated with both an interquartile (25th to 75th percentile) and a 5th- to 95th-percentile increase in the source estimate was computed for lag days 0 to 5 for each of the source apportionment analyses. Again, this consistent mortality analysis approach across source apportionments allowed a direct comparison of the daily mortality effect estimates across the various source apportionment analyses in each city.

Finally, we evaluated the size and significance of the additional variability introduced to the PM–mortality, time-series analysis by variations in the source apportionment process across groups and methods, consistent with the primary goal of this workshop. To this end, the various source apportionments’ resulting mean mass contributions and estimated percent excess deaths per 5th- to 95th-percentile increment increase by source-apportioned PM_2.5_ were intercompared and then analyzed (within each city) by analysis of variance (ANOVA) and a GLM. This allowed us to compare variations in model estimates that were due to “between-source” versus “within-source” (i.e., variation due to different analyses).

## Results

### Source apportionment intercomparisons.

**Figure 1:**
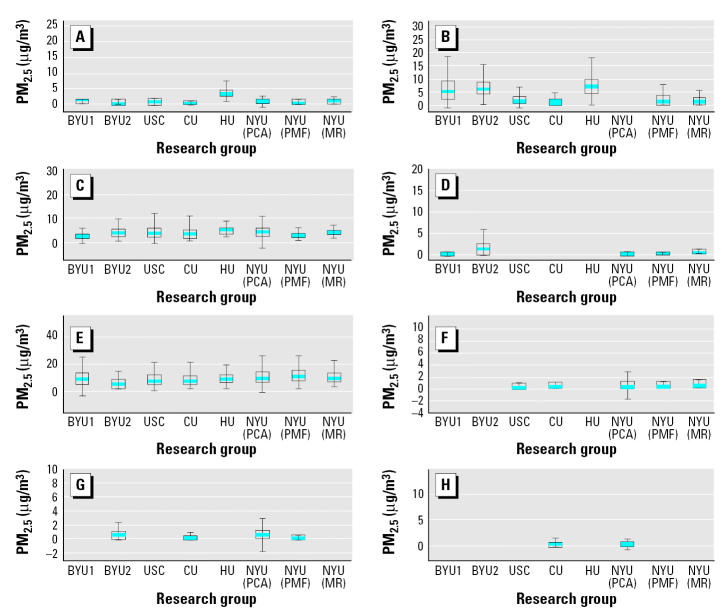
Mean, interquartile range (box), and range (maximum–minimum) of mass impacts predicted by each research group’s source apportionment analysis of the Washington PM_2.5_ data set. MR, multiple regression. (*A*) Soil; (*B*) nitrates; (*C*) traffic; (*D*) wood burning; (*E*) secondary SO_4_; (*F*) residual oil; (*G*) sea salt; (*H*) incinerator.

**Figure 2:**
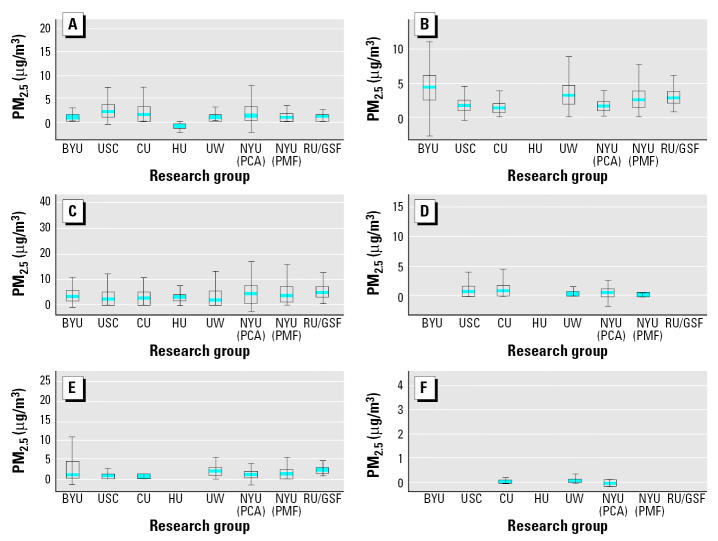
Mean, interquartile range (box), and range (maximum–minimum) of mass impacts predicted by each research group’s source apportionment analysis of the Phoenix PM_2.5_ data. (*A*) Soil; (*B*) secondary SO_4_; (*C*) traffic; (*D*) metals/industry/smelter; (*E*) vegetation/wood burning; (*F*) sea salt.

As described in Hopke et al. (in press), the various source apportionment analyses from each of the participating research groups were inter-compared in two ways: *a*) by comparing the mass contributions attributed to each of the sources; and *b*) by calculating the correlation coefficients between the source contributions from PM_2.5_ from the various groups within source groups. The solutions of the various groups were compared with each other on an equal basis, because an accepted “gold standard” method does not exist at this time for source apportionment. Table 2 notes the source apportionment analyses performed on these data sets. Figures 1 and 2 present the means and distributions of the resulting PM_2.5_ mass source apportionment for each source category in Washington and Phoenix, respectively. Most groups were able to identify the same major sources in their source apportionment analyses of the trace constituent data. However, not all sources were identified by all researchers; some groups did not provide impacts for all possible source categories. Two researchers from BYU contributed separate analyses: Eatough (BYU1) and Christensen (BYU2). In this plot, when researchers broke out the source impacts differently from other researchers (e.g., when secondary sulfates were broken into sulfates 1 and sulfates 2, or traffic was subdivided into categories, such as diesel vs. gasoline fueled motor vehicles), the results have been grouped to provide more directly comparable totals. The mass apportionment uncertainties included in Figures 1 and 2 visually indicate an overall consistency in impacts by source category, as they provide confidence intervals (CIs) that overlap across analyses of the various groups, especially for the larger mass contributors. To be more quantitative, we conducted an ANOVA F-test of the within-source versus between-source variations for each of the major source categories in Figures 1 and 2. The results indicated significantly greater variability (*p* < 0.001) across source categories than across investigators/methods (i.e., investigator/method variations were small compared with source-to-source variations). Overall, these plots and statistical analyses indicate that, although the estimated mass impact results vary across analyses and not all sources were identified by all investigators (especially in the case of the smaller mass impact sources), there is both qualitative and quantitative consistency in the major PM_2.5_ contributing sources identified and their mass impacts across the independent analyses of these data by the various research groups and apportionment methods.

**Figure 3:**
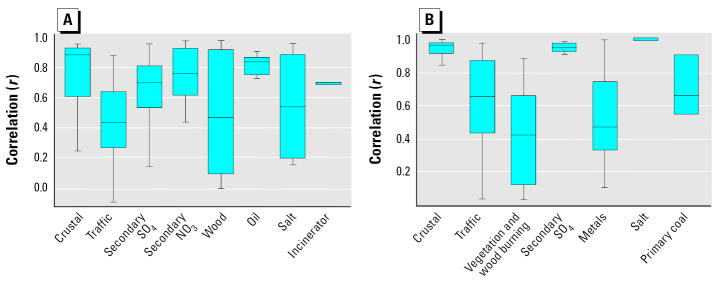
Box and whisker plots of the distributions of temporal correlation coefficients (*r*) between all possible pairs of similar source contributions resolved for (*A*) Washington and (*B*) Phoenix.

Because these apportionment results were to be applied in time-series analyses, another evaluation of the consistency of the source apportionments across research groups and apportionment methods was conducted. Variability was examined in the paired correlations of estimated daily source apportionment mass contributions in the various analyses over time and within each city. As shown in Figure 3A for Washington and Figure 3B for Phoenix, the sulfate-containing, crustal, and nitrate components exhibited among the highest mean intercorrelations across the various research groups in these cities. Among the chief PM_2.5_ mass contributors (Figures 1 and 2), the weakest cross-analyses correlations in Figure 3 were usually found for the sources with the greatest uncertainty in their composition (i.e., lacking unique constituents for unique identification), notably, traffic and wood burning in Washington, and wood burning and metals in Phoenix.

### Time-series mortality effect estimate intercomparisons.

**Figure 4:**
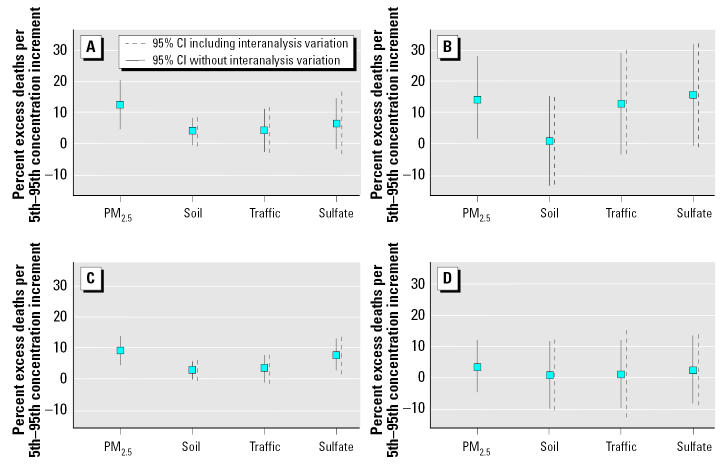
Mean RR estimates and 95% CIs for each major source category in Washington (*A*) cardiovascular and (*C*) total nonaccidental mortality, and Phoenix (*B*) cardiovascular and (*D*) total nonaccidental mortality for the overall workshop estimate, with source apportionment interanalysis variation excluded and with the interanalysis variation included.

The source apportionment results for each group were combined with the mortality data in Washington and Phoenix, and time-series mortality regressions were then run (Ito et al., in press; Mar et al., in press). Figure 4 displays the resulting mean RR estimates and 95% CIs of cardiovascular (CV) and total daily mortality for each major source category identified in Washington and Phoenix for the overall workshop estimate, with source apportionment interanalysis variation excluded and interanalysis variation included. Results were derived using the lag of maximum association in each analysis. It is clear from the comparisons that the variability introduced by the uncertainty of the across-source apportionment groups and analyses is small, relative to the overall uncertainty of these estimates. In quantitative terms, the percent increase in the uncertainty (i.e., in the CI) for the mortality RR of each displayed source category in Washington added by the interanalysis variability was as follows: soil (23% for CV, 18% for total); traffic (12% for CV, 16% for total); and sulfate (25% for CV, 26% for total). In the Phoenix mortality analyses, the percent increase in the uncertainty (i.e., in the CI) for the mortality RR of each displayed source category added by the interanalysis variability was as follows: soil (4% for CV, 7% for total); traffic (6% for CV, 33% for total); and sulfate (7% for CV, 5% for total). Thus, while the uncertainty added by the differences in source apportionments varies from source to source in these cases, the overall average increase is about 15%, which suggests that the error added by variability in source apportionment approach is quite small relative to the baseline uncertainty inherently associated with making these time-series pollution RR estimates.

The between-source variation in these daily mortality RRs was also compared with within-source variations (variation due to different analyses). As shown in Table 3, significantly larger variation was found between sources than between research groups in reported RRs (*p* < 0.001) using an ANOVA (in a GLM) of the individual investigator estimates and variances (for each death category in each city) (Ito et al., in press; Mar et al., in press). In the GLM, between-group variation was a nonsignificant predictor for both death categories in both cities (with *p*-values ranging from 0.38 to 0.65 for between-group differences), whereas the between-source variation was a statistically significant predictor of RR in both cities and death categories (*p* < 0.001). Overall, these results indicate that *a*) variations in choice of research group or source apportionment method have only a small effect on variations in the RR estimates for identified sources, relative to the variations in RR caused by different source components and the mortality regression process, and *b*) researcher variations in source apportionment applications should not be a barrier to comparing the source-specific PM_2.5_ RRs.

The size of the source-specific RR estimates from these analyses can also be compared with other published source-category effect estimates, although very few are available currently. The most consistently significant category was secondary sulfates, which have been widely examined before in the published literature. In this case, the total mortality RR estimates for the secondary sulfate component were 5.2% change per 10 μg/m^3^ in Phoenix and 3.8% per 10 μg/m^3^ in Washington. This is somewhat larger than the sulfate-dominated coal component reported by Laden et al. (2000), but much smaller than that derived from Ozkaynak and Thurston (1987). Their research indicated 8% per 10 μg/m^3^ for this component, but that study was of annual mortality associated with long-term exposures, rather than the daily mortality considered here. It is interesting, however, that the Washington component estimate from this work (3.8% per 10 μg/m^3^ for the sulfate component) is very close to the sulfate-related coal component value derived by Laden and colleagues for Boston, Massachusetts (2.8%). Motor vehicles, another component that approached significance in this work, yielded 0.9% per 10 μg/m^3^ RR in Phoenix, and 4.2% in Washington. These results are similar to the 3.4% per 10 μg/m^3^ found by Laden and colleagues (2000), and the 2% per 10 μg/m^3^ derived from the work of Ozkaynak and Thurston (1987). Thus, these source-specific estimates appear reasonable when compared with the limited source-specific mortality analyses done in the past, but much more work of this type must be done before broad-based comparisons with the RR results from this workshop are possible.

## Discussion and Conclusions

With regard to the PM_2.5_ mass apportionments, the findings of this intercomparison among results from some of the leading source apportionment research groups indicate that the same major source types (those that contribute most of the PM_2.5_ mass at each site), with similar elemental makeups (i.e., key tracers), are consistently identified by different groups in each city. Methods generally yielded the most consistent results (i.e., the highest correlations across groups over time) for sources with the most definable (unique) tracers or combinations of tracers in each city. In Washington, soil, secondary sulfate and nitrate, oil burning, and incineration were most unambiguously identified by various methods; wood burning, salt, and traffic were less well correlated across analyses. In Phoenix, soil, traffic, secondary sulfate, and sea spray were most highly correlated across analyses; wood and vegetative burning, metals industry particles, and coal fly ash were less well correlated. Based on the relative sizes of these intergroup intercorrelations for each of the source types in these two cities, the soil-, sulfate-, residual oil-, and salt-associated mass components were generally seen to be most unambiguously identified by the various source apportionment methods, while vegetative burning and traffic were less well correlated across groups. However, the source mass impacts predicted for the various source categories were generally not significantly different from one another across the research groups, indicating consistency in the source apportionment results. The addition of further tracers/analyses may be required to improve the consistency of the less well-discriminated sources. For example, the measurement of low-volatility organic compounds has been suggested as one way to better discern traffic-related PM components (Schauer et al. 1996; Schauer and Cass 2000). Overall, however, although there are no gold standard correct answers for the source identification and apportionments in the real-world data sets considered in this workshop, the apportionment consistency in the largest PM_2.5_ source contributors across researchers in these cities, often using differing statistical methods, indicates reliability in the source apportionment approach.

With regard to the health effects apportionments to the different source components of PM_2.5_, the between-source variation in daily mortality RR was significantly larger than the between-research group variation in reported RRs. Thus, analysis-to-analysis variability in the source apportionments was small compared with the overall uncertainty in the mortality RR estimates. In addition, between-group variation in RR estimates was nonsignificant, whereas the between-source type variation was statistically significant. This result indicates that variations in choice of research group or source apportionment method have only a small effect on variations in the RR estimates, relative to the variations in RR caused by different source components. Indeed, in mortality categories where significant PM_2.5_ mass–daily mortality associations were detected in these cities (e.g., for cardiovascular deaths in both cities), most source categories were non-significant contributors. However, the most strongly associated source categories showed statistically significant contributions. Across these two cities, the most consistently associated PM_2.5_ source category was sulfate-associated mass. The source RR estimates generally had overlapping confidence bands, indicating that larger numbers of observations will be required in each of these cities to have enough power to significantly differentiate the impacts of the various source impacts. The overall source-specific RR estimates derived in this work appeared reasonable when compared with the limited source-specific mortality analyses published in the past, but many more source apportionment–mortality analyses of this type must be done before broad-based comparisons with the source-specific RR results from this workshop are possible.

Overall, the results of this intercomparison of the health effects apportionments found that variations in PM source apportionment research group or method introduced relatively little uncertainty into the evaluation of differences in PM toxicity on a source-specific basis, adding an average of only approximately 15% to the overall source-specific mortality RR uncertainties. Variations in these apportionment modeling choices do not prevent the consistent discernment of variations in the relative strengths of source-specific PM_2.5_ mortality associations. However, the uncertainty added by the source apportionment estimation suggests that longer data records may be required for significant effects to be detectable in source-specific analyses than for PM_2.5_. The conduct of daily speciation sampling (rather than every third day) in major U.S. cities would be one way to rapidly improve the power of future source apportioned PM time-series health effects analyses. Daily sampling would also better clarify the potentially differing distributed-lag natures of the various source-specific impacts identified in this workshop. Although further research and the possible addition of more key tracers to the speciation of PM_2.5_ are needed to better characterize ambient tracer profiles for sources with less well-defined compositional characteristics (e.g., for vegetative burning and traffic), the results of this workshop indicate that present-day PM_2.5_ source apportionment methods can provide valuable insights into the source components that contribute most to PM_2.5_–health effects associations.

## Figures and Tables

**Table 1:** Summary of workshop goals and participating research institutions.

Workshop goals	Participating research institutions
To bring together key researchers to assess the reliability of source apportionment–health effects methods by analyzing daily mortality with existing PM_2.5_ data sets similar to those now being collected by the U.S. EPA Specialization Network.	Brigham Young University (BYU) Clarkson University (CU) Harvard University (HU) New York University (NYU) University of Rochester and GSF (UR/GSF)
To identify key future research needs for source apportionment– health effects evaluation.	University of Southern California (USC) University of Washington (UW)

GSF, German National Research Center for Environment and Health.

**Table 2:** Summary of the source apportionment analyses performed by each participating group.

Research institutions	Phoenix, AZ	Washington, DC
BYU	Unmix	Unmix, iterated, confirmatory FA
CU	PMF2 and expanded model (ME)	PMF2
HU	Target rotated PCA	Target rotated PCA
NYU	PMF, APCA	PMF, APCA, single-elemental multiple regression
UR/GSF	APCA	
USC	Unmix	Unmix
UW	PMF	

**Table 3:** ANOVA analysis of source-specific mortality RR estimates.

Mortality category	ANOVA *p*-value	Source category variance (%)	Research group variance (%)
Washington CV	< 0.001	47.5	9.5
Washington total	< 0.001	80.0	2.6
Phoenix CV	< 0.001	76.3	4.5
Phoenix total	< 0.001	64.8	6.3
